# Supplementary material for: Causal contributions of left inferior and medial frontal cortex to semantic and executive control
Source: Commun Biol. 2025 Sep 12;8:1343. doi: 10.1038/s42003-025-08848-5 (PMC12432203; doi:10.1038/s42003-025-08848-5)
Supplement: Supplementary file 1 — Supplementary Material [file 42003_2025_8848_MOESM1_ESM.pdf]

Supplementary Materials

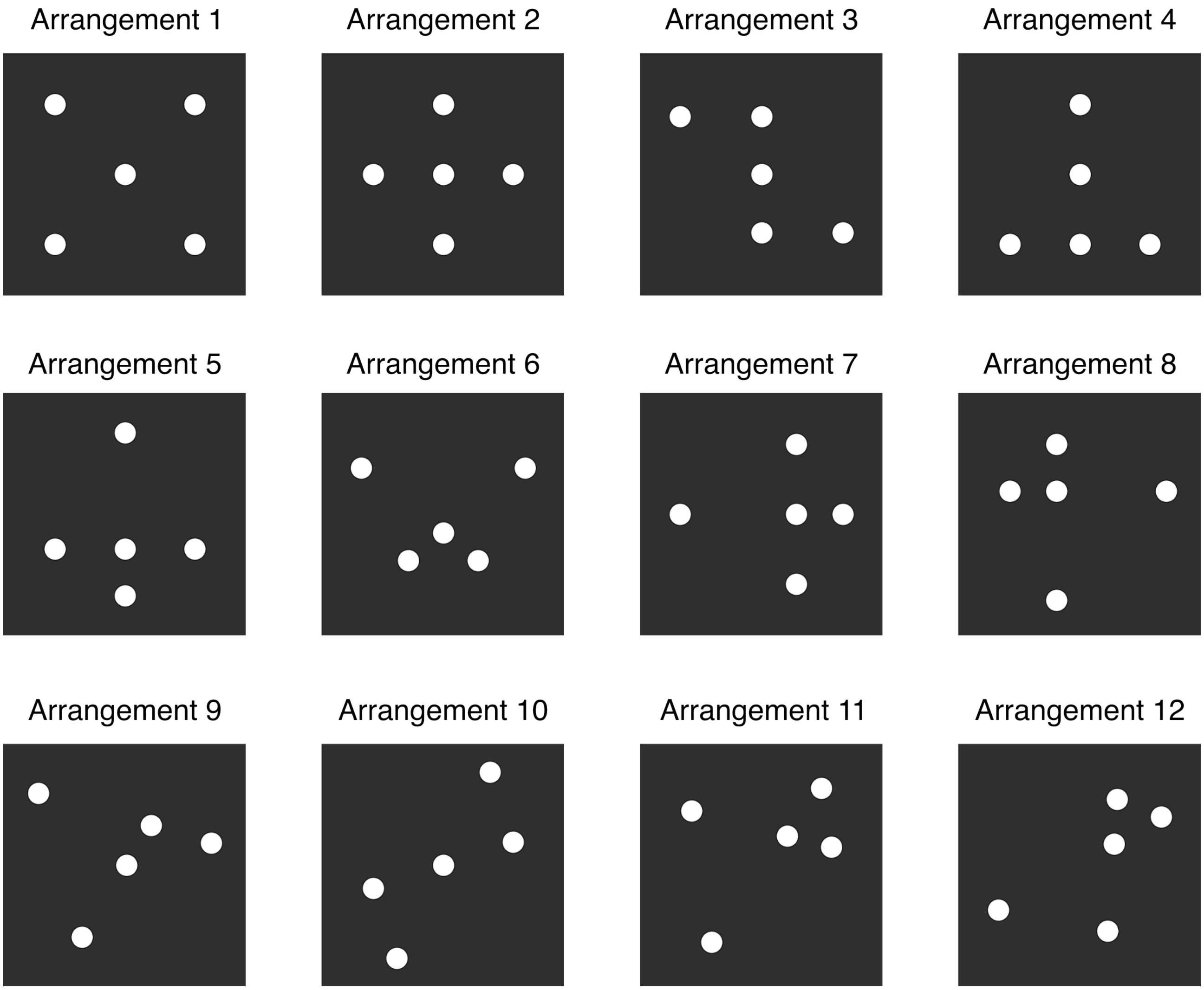

Figure S1. Dot configurations for five-point task. We developed a computerized version of the five-point task and designed 11 additional dot arrangements so that participants had to complete three unique dot arrangements per session.

Table S1. Descriptive statistics of stimulus lists for picture naming

Stimulus list	Word frequency (frequency class)	Word length in syllables	Name agreement	Visual complexity (rating scale 1-5)
1	13.53 (2.14)	2.09 (0.74)	0.64 (0.58)	2.75 (0.55)
2	13.39 (2.18)	2.09 (0.74)	0.61 (0.56)	2.75 (0.53)
3	13.51 (2.31)	2.09 (0.74)	0.63 (0.51)	2.78 (0.54)
4	13.43 (2.30)	2.09 (0.74)	0.61 (0.57)	2.73 (0.52)

Note. For each parameter, mean and standard deviation are given. Word frequencies in the form of frequency class were derived from the database Wortschatz Leipzig (https://corpora.uni-leipzig.de/de?corpusId=deu_news_2022).

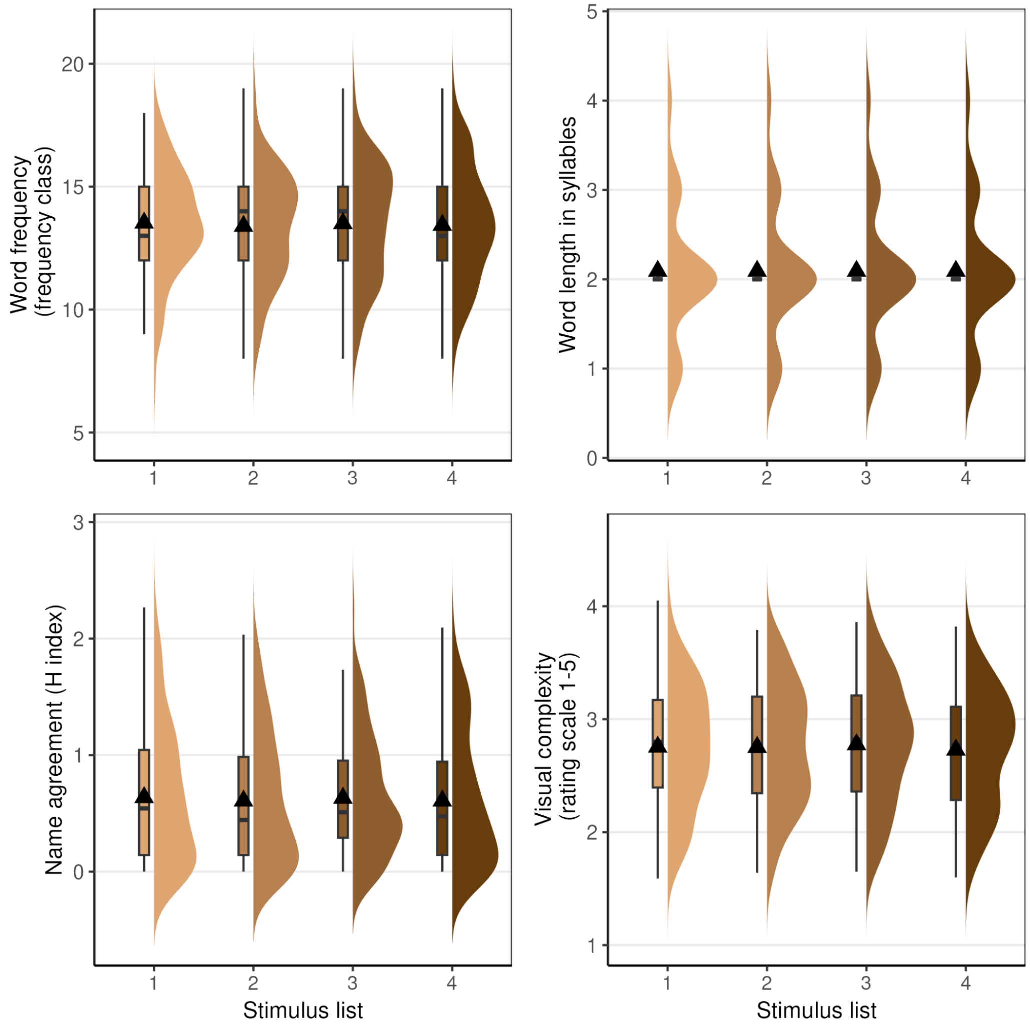

Figure S2. Descriptive statistics of stimulus lists for picture naming. Boxplots show first quartile, median, and third quartile, with the box highlighting the interquartile range. Triangles indicate mean values.

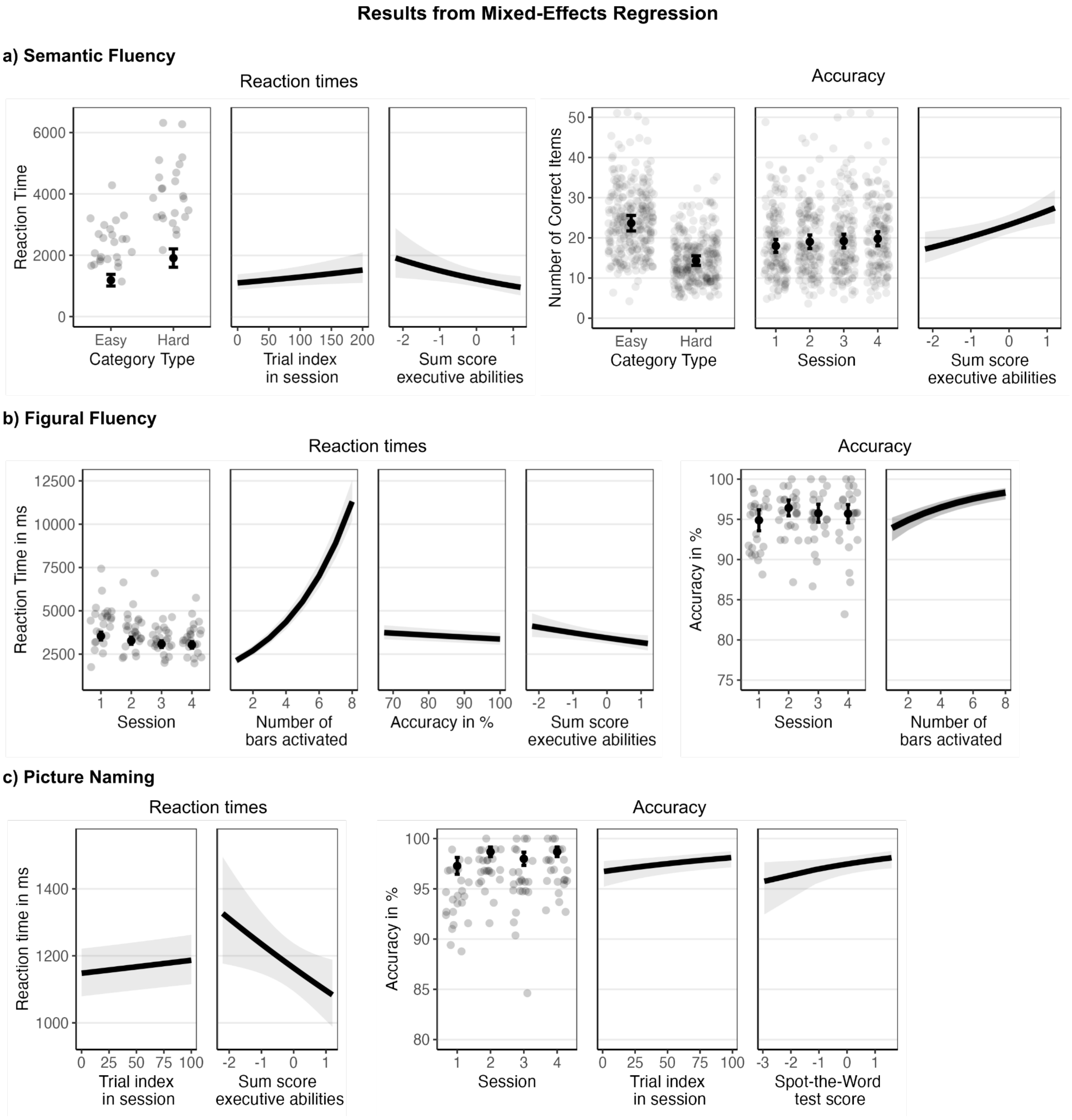

Figure S3. Results from mixed-effects regression. Panels a, b, and c show significant main effects for other predictors than stimulation condition for reaction times and accuracy in each task. For categorical predictors, raw data are plotted in the background. Note that as LMM predicted values represent adjusted, model-based estimates, they might diverge from naive group averages.

Table S2. Reaction times: Results from mixed-effects regression for each task.

	Semantic Fluency				Figural Fluency				Picture Naming			
Predictors	*Estimates (CI)*	*Statistic*	p_{uncorr}	p_{FDR}	*Estimates (CI)*	*Statistic*	p_{uncorr}	p_{FDR}	*Estimates (CI)*	*Statistic*	p_{uncorr}	p_{FDR}
Target [IFG vs. Sham]	0.09 (0.02 – 0.17)	2.49	**0.015**	**0.024**	0.04 (0.01 – 0.08)	2.32	**0.021**	**0.046**	0.01 (-0.01 – 0.02)	1.00	0.318	0.381
Target [PreSMA vs. Sham]	0.12 (0.05 – 0.20)	3.29	**0.001**	**0.007**	0.04 (0.00 – 0.08)	2.16	**0.031**	**0.046**	-0.01 (-0.02 – 0.00)	-1.49	0.135	0.203
Target [Dual site vs. Sham]	0.11 (0.03 – 0.18)	2.81	**0.006**	**0.015**	0.04 (-0.01 – 0.08)	1.73	0.084	0.084	-0.01 (-0.03 – 0.00)	-1.68	0.093	0.185
Session [2 vs. 1]	0.01 (-0.06 – 0.09)	0.37	0.713		-0.08 (-0.12 – -0.04)	-3.87	**<0.001**		0.01 (-0.01 – 0.02)	0.76	0.447	
Session [3 vs. 1]	0.03 (-0.05 – 0.10)	0.67	0.500		-0.14 (-0.18 – -0.10)	-6.75	**<0.001**		0.00 (-0.01 – 0.02)	0.36	0.719	
Session [4 vs. 1]	0.03 (-0.04 – 0.11)	0.84	0.401		-0.15 (-0.20 – -0.11)	-7.05	**<0.001**		0.00 (-0.01 – 0.02)	0.28	0.779	
Category type [Hard vs. Easy]	0.48 (0.37 – 0.59)	8.49	**<0.001**									
Task order [SF first vs. FF first]	-0.27 (-0.56 – 0.02)	-1.94	0.068		-0.08 (-0.19 – 0.04)	-1.41	0.172		-0.06 (-0.14 – 0.03)	-1.31	0.205	
Trial Index	0.00 (0.00 – 0.00)	2.24	**0.025**		0.00 (-0.00 – 0.00)	1.93	0.053		0.00 (0.00 – 0.00)	3.47	**0.001**	
Spot-the-Word test	-0.07 (-0.22 – 0.07)	-1.03	0.318						-0.02 (-0.06 – 0.02)	-0.97	0.344	
Executive score	-0.20 (-0.38 – -0.02)	-2.36	**0.028**		-0.08 (-0.15 – -0.01)	-2.38	**0.026**		-0.06 (-0.11 – -0.01)	-2.30	**0.032**	
Stimulus list [b vs. a]					0.06 (0.00 – 0.12)	2.11	**0.047**					

Predictors	Estimates (CI)	Statistic	p	Estimates (CI)	Statistic	p	Estimates (CI)	Statistic	p
Stimulus list [c vs. a]				0.04 (-0.02 – 0.11)	1.40	0.175			
Stimulus list [d vs. a]				0.03 (-0.04 – 0.10)	0.97	0.340			
Number of bars				0.24 (0.23 – 0.24)	77.00	**<0.001**			
Accuracy				-0.32 (-0.50 – -0.14)	-3.47	**0.001**	-0.00 (-0.00 – 0.00)	-0.57	0.567
Marginal / Conditional R^2	0.058 / 0.190			0.469 / 0.625			0.045 / 0.436		

Note. Significant effects are marked in bold. Uncorrected p-values with underscore signify the threshold value for FDR correction. CI: Confidence interval.

Table S3. Accuracy: Results from mixed-effects regression for each task.

	Semantic Fluency				Figural Fluency				Picture Naming			
Predictors	*Incidence Rate Ratios (CI)*	*Statistic*	p_{uncorr}	p_{FDR}	*Odds Ratios (CI)*	*Statistic*	p_{uncorr}	p_{FDR}	*Odds Ratios (CI)*	*Statistic*	p_{uncorr}	p_{FDR}
Target [IFG vs. Sham]	0.97 (0.91 – 1.04)	-0.83	0.405	0.506	1.17 (0.90 – 1.50)	1.19	0.236	0.337	1.00 (0.72 – 1.38)	-0.01	0.337	0.989
Target [PreSMA vs. Sham]	0.96 (0.90 – 1.02)	-1.25	0.212	0.506	0.96 (0.75 – 1.23)	-0.34	0.736	0.818	0.95 (0.69 – 1.31)	-0.31	0.818	0.754
Target [Dual site vs. Sham]	0.94 (0.88 – 1.00)	-1.84	0.066	0.330	0.79 (0.62 – 1.00)	-1.99	**0.047**	0.117	0.81 (0.59 – 1.10)	-1.34	0.117	0.180
Session [2 vs. 1]	1.06 (0.99 – 1.13)	1.60	0.110		1.45 (1.13 – 1.86)	2.97	**0.003**		2.09 (1.52 – 2.87)	4.51	**<0.001**	
Session [3 vs. 1]	1.07 (1.00 – 1.14)	1.87	0.062		1.22 (0.96 – 1.54)	1.64	0.102		1.36 (1.02 – 1.81)	2.08	**0.037**	
Session [4 vs. 1]	1.10 (1.03 – 1.18)	2.73	**0.006**		1.20 (0.95 – 1.52)	1.53	0.126		2.09 (1.52 – 2.86)	4.56	**<0.001**	

Category type [Hard vs. Easy]	0.61 (0.58 – 0.64)	-20.35	**<0.001**						
Spot-the-Word test	1.00 (0.93 – 1.08)	-0.02	0.981				1.20 (1.01 – 1.42)	2.10	**0.035**
Executive score	1.15 (1.05 – 1.26)	2.96	**0.003**	0.93 (0.70 – 1.24)	-0.48	0.634	1.06 (0.86 – 1.29)	0.53	0.595
Number of bars				1.21 (1.13 – 1.29)	5.76	**<0.001**			
Trial Index				1.00 (1.00 – 1.00)	-0.12	0.905	1.01 (1.00 – 1.01)	2.64	**0.008**
Marginal / Conditional R^2	0.410 / 0.575			0.034 / 0.109			0.030 / 0.396		

Note. Significant effects are marked in bold. Uncorrected p-values with underscore signify the threshold value for FDR correction. CI: Confidence interval.

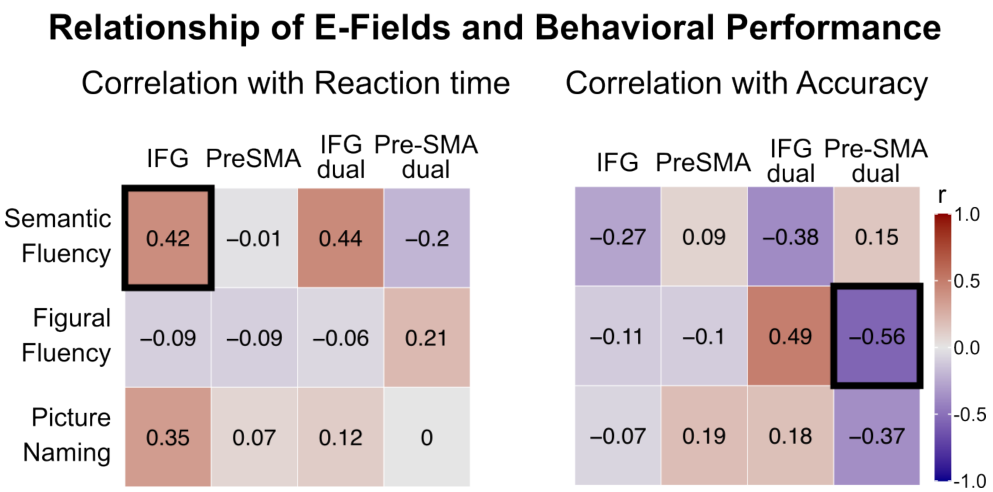

Figure S4. Correlation matrices for all tasks and stimulation sessions for reaction time and accuracy, respectively. Significant results after FDR correction are marked in bold. Note that for the dual-site stimulation, results are based on a regression model using both e-fields as predictors and values thus represent individual standardized coefficients from multiple linear regression rather than correlation coefficients.

Table S4. Comparison of e-field values in subregions of cortical stimulation targets.

	E-Field 95th Percentile			
Predictors	*Estimates (CI)*	*Statistic*	p_{uncorr}	p_{FDR}
ROI [BA44 vs. BA45]	-5.65<br>(-8.23 – -3.07)	-4.33	**<0.001**	**<0.001**
ROI [pMFG vs. BA45]	-5.11<br>(-7.69 – -2.53)	-3.91	**<0.001**	**<0.001**
ROI [left pre-SMA vs. BA45]	-19.17<br>(-21.75 – -16.60)	-14.68	**<0.001**	**<0.001**
ROI [right pre-SMA vs. BA45]	-22.52<br>(-25.10 – -19.94)	-17.23	**<0.001**	**<0.001**
Session [Pre-SMA vs. IFG]	-22.55<br>(-25.30 – -19.81)	-17.01	**<0.001**	**<0.001**
ROI [BA44 vs. BA45] x Session [Pre-SMA vs. IFG]	6.43<br>(1.27 – 11.58)	2.46	**<u>0.015</u>**	**0.015**
ROI [pMFG vs. BA45] x Session [Pre-SMA vs. IFG]	19.55<br>(14.39 – 24.70)	7.48	**<0.001**	**<0.001**
ROI [left pre-SMA vs. BA45] x Session [Pre-SMA vs. IFG]	88.86<br>(83.71 – 94.02)	34.00	**<0.001**	**<0.001**
ROI [right Pre-SMA vs. BA45] x Session [Pre-SMA vs. IFG]	84.61<br>(79.46 – 89.77)	32.38	**<0.001**	**<0.001**
Marginal R^2 / Conditional R^2	0.849 / 0.940			

Note. Significant effects are marked in bold. Uncorrected p-values with underscore signify the threshold value for FDR correction. CI: Confidence interval.

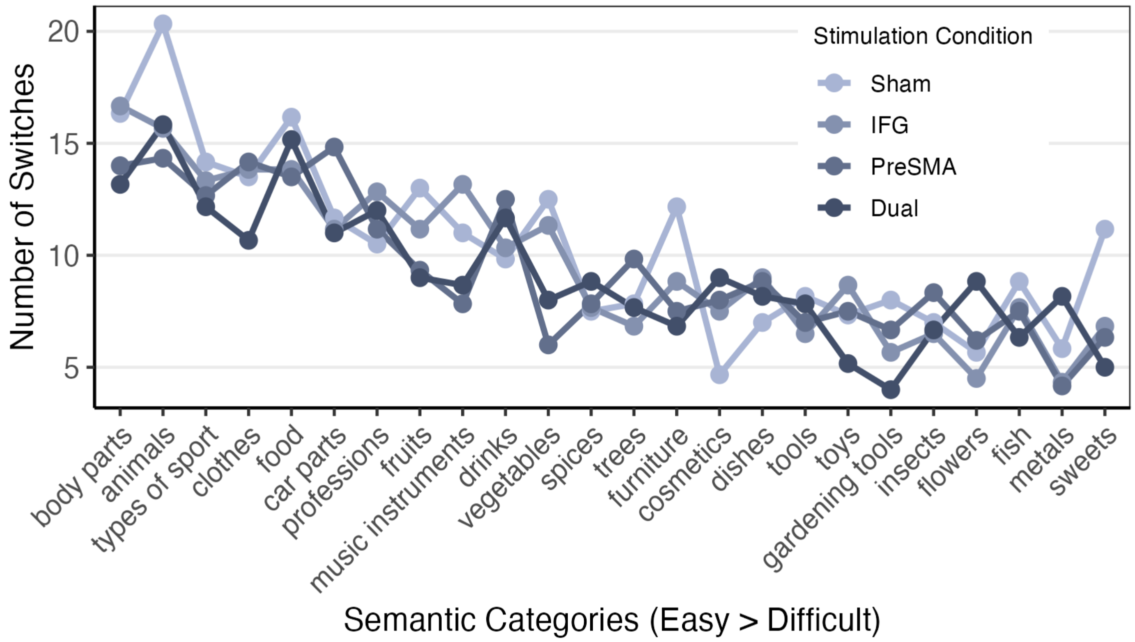

Figure S5. Number of switches for each semantic category and stimulation condition, ordered according to increasing difficulty.

Table S5. Results from mixed-effects regression for number of switches during semantic fluency

	Number of switches per category			
Predictors	*Incidence Rate Ratios (CI)*	*Statistic*	p_{uncorr}	p_{FDR}
Target [IFG vs. Sham]	0.93 (0.86 – 1.01)	-1.73	0.084	0.143
Target [PreSMA vs. Sham]	0.90 (0.83 – 0.98)	-2.44	**0.015**	**0.039**
Target [Dual site vs. Sham]	0.87 (0.81 – 0.95)	-3.24	**0.001**	**0.007**
Session [2 vs. 1]	1.06 (0.98 – 1.15)	1.40	0.163	
Session [3 vs. 1]	1.06 (0.97 – 1.15)	1.28	0.200	
Session [4 vs. 1]	1.08 (1.00 – 1.18)	1.94	0.053	
Category type [Hard vs. Easy]	0.60 (0.52 – 0.69)	-7.28	**<0.001**	
Marginal R^2 / Conditional R^2	0.255 / 0.572			

Note. Reported p-values of stimulation conditions are FDR-corrected. Significant effects are marked in bold. CI: Confidence interval.
