# Supplementary material for: Causal contributions of left inferior and medial frontal cortex to semantic and executive control
Source: Commun Biol. 2025 Sep 12;8:1343. doi: 10.1038/s42003-025-08848-5 (PMC12432203; doi:10.1038/s42003-025-08848-5)
Supplement: Supplementary file 2 — Reporting Summary [file 42003_2025_8848_MOESM2_ESM.pdf]

nature portfolio

Corresponding author(s): Sandra Martin
Last updated by author(s): May 9, 2025

Reporting Summary

Nature Portfolio wishes to improve the reproducibility of the work that we publish. This form provides structure for consistency and transparency in reporting. For further information on Nature Portfolio policies, see our Editorial Policies and the Editorial Policy Checklist.

Statistics

For all statistical analyses, confirm that the following items are present in the figure legend, table legend, main text, or Methods section.

n/a	Confirmed	
☐	☒	The exact sample size (*n*) for each experimental group/condition, given as a discrete number and unit of measurement
☐	☒	A statement on whether measurements were taken from distinct samples or whether the same sample was measured repeatedly
☐	☒	The statistical test(s) used AND whether they are one- or two-sided<br>*Only common tests should be described solely by name; describe more complex techniques in the Methods section.*
☐	☒	A description of all covariates tested
☐	☒	A description of any assumptions or corrections, such as tests of normality and adjustment for multiple comparisons
☐	☒	A full description of the statistical parameters including central tendency (e.g. means) or other basic estimates (e.g. regression coefficient) AND variation (e.g. standard deviation) or associated estimates of uncertainty (e.g. confidence intervals)
☐	☒	For null hypothesis testing, the test statistic (e.g. F, t, r) with confidence intervals, effect sizes, degrees of freedom and P value noted<br>*Give P values as exact values whenever suitable.*
☒	☐	For Bayesian analysis, information on the choice of priors and Markov chain Monte Carlo settings
☐	☒	For hierarchical and complex designs, identification of the appropriate level for tests and full reporting of outcomes
☐	☒	Estimates of effect sizes (e.g. Cohen's d, Pearson's r), indicating how they were calculated

Our web collection on statistics for biologists contains articles on many of the points above.

Software and code

Policy information about availability of computer code

Data collection	All tasks were programmed and presented with Psychopy version 2021.2.3.
Data analysis	Statistical models were conducted using R v.4.4.1 (R Core Team, 2024) with the lme4 package (Bates et al., 2015) for mixed-effects models and the performance package (Lüdecke et al., 2021) for model comparisons. Post-hoc multiple comparisons were performed using the emmeans package (Lenth, 2020) to determine effects between individual factor levels other than the reference levels, with FDR correction applied. Plots were generated using ggplot2 (Wickham, 2016) and ggeffects (Lüdecke, 2018), and model output via sjPlot (Lüdecke, 2021). We performed post-hoc e-field simulations using SimNIBS v.4.0.0 (Thielscher et al., 2015). Recordings from the semantic fluency and the picture naming tasks were transcribed by three native German raters who annotated reaction times with Praat (Boersma & van Heuven, 2001).

For manuscripts utilizing custom algorithms or software that are central to the research but not yet described in published literature, software must be made available to editors and reviewers. We strongly encourage code deposition in a community repository (e.g. GitHub). See the Nature Portfolio guidelines for submitting code & software for further information.

Data

Policy information about availability of data

All manuscripts must include a data availability statement. This statement should provide the following information, where applicable:

- Accession codes, unique identifiers, or web links for publicly available datasets
- A description of any restrictions on data availability
- For clinical datasets or third party data, please ensure that the statement adheres to our policy

Data and analysis scripts are available in our OSF repository https://osf.io/q5kam/.

Research involving human participants, their data, or biological material

Policy information about studies with human participants or human data. See also policy information about sex, gender (identity/presentation), and sexual orientation and race, ethnicity and racism.

Reporting on sex and gender	We tested 24 healthy adults of whom 13 identified as female. We did not have any gender-specific hypotheses nor tested such hypotheses and only aimed to balance gender in our sample.
Reporting on race, ethnicity, or other socially relevant groupings	We did not assess grouping variables on race, ethnicity, or other socially relevant groupings and did also not have hypotheses for these variables.
Population characteristics	We tested 24 healthy adults (M = 30.00, SD = 5.32, range: 20–40 years, 13 female), who were right-handed, native German speakers, and had no contraindication to MRI and TMS.
Recruitment	Participants were recruited via the participant database of the MPI for Human Cognitive and Brain Sciences.
Ethics oversight	The study was approved by the local ethics committee of the Medical Faculty at Leipzig University.

Note that full information on the approval of the study protocol must also be provided in the manuscript.

Field-specific reporting

Please select the one below that is the best fit for your research. If you are not sure, read the appropriate sections before making your selection.

☐ Life sciences ☒ Behavioural & social sciences ☐ Ecological, evolutionary & environmental sciences

For a reference copy of the document with all sections, see nature.com/documents/nr-reporting-summary-flat.pdf

Behavioural & social sciences study design

All studies must disclose on these points even when the disclosure is negative.

Study description	We conducted an experimental quantitative study with a repeated measures within-subjects design.
Research sample	We tested 24 healthy adults (M = 30.00, SD = 5.32, range: 20–40 years, 13 female), who were right-handed, native German speakers, and had no contraindication to MRI and TMS. We tested healthy young participants since we did not aim to test for age-specific effects and did not intend to test clinical populations.
Sampling strategy	Participants were randomly sampled from our in-house database after being eligible to TMS and MRI and consenting to participation. We included 24 participants which is above the average for a repeated-measures design with four sessions in studies applying non-invasive brain stimulation. We did run an a priori power analysis due to the lack of comparable studies and effect sizes. However, we included a number divisable by four to have a counterbalanced design for order of stimulation conditions.
Data collection	We employed a repeated-measures within-subjects design with four sessions per participant, which were separated by at least one week. At the beginning of each session, participants (re-)familiarized themselves with the task. They then received offline rTMS stimulation and subsequently performed the experiment (Fig. 1A). The study comprised four stimulation conditions: IFG, pre-SMA, dual TMS (IFG first, followed by pre-SMA stimulation), and sham stimulation (Fig. 1B). The order of these conditions was counterbalanced across subjects. Participants were blinded to the type of stimulation.<br>Reaction times and accuracy data were collected via Psychopy. In the semantic fluency and picture naming tasks, participants were recorded and recordings were later transcribed and analysed regarding accuracy and reaction times.
Timing	Data were collected between August 2022 and January 2023.
Data exclusions	We did not exclude full data sets. To exclude excessive outlier values from RTs, we employed a lenient approach where for each participant, session, task, and stimulus item (category, figure), RTs at least three SDs above the mean were excluded. This procedure removed 3.6% of all trials.

Non-participation	No participants dropped out of the study.
Randomization	The order of stimulation conditions (sham, IFG, preSMA, dual site) was counterbalanced across participants. This was done manually to control for a fully balanced design.

Reporting for specific materials, systems and methods

We require information from authors about some types of materials, experimental systems and methods used in many studies. Here, indicate whether each material, system or method listed is relevant to your study. If you are not sure if a list item applies to your research, read the appropriate section before selecting a response.

Materials & experimental systems

n/a	Involved in the study
☒	☐ Antibodies
☒	☐ Eukaryotic cell lines
☒	☐ Palaeontology and archaeology
☒	☐ Animals and other organisms
☒	☐ Clinical data
☒	☐ Dual use research of concern
☒	☐ Plants

Methods

n/a	Involved in the study
☒	☐ ChIP-seq
☒	☐ Flow cytometry
☐	☐ MRI-based neuroimaging

Plants

Seed stocks	*Report on the source of all seed stocks or other plant material used. If applicable, state the seed stock centre and catalogue number. If plant specimens were collected from the field, describe the collection location, date and sampling procedures.*
Novel plant genotypes	*Describe the methods by which all novel plant genotypes were produced. This includes those generated by transgenic approaches, gene editing, chemical/radiation-based mutagenesis and hybridization. For transgenic lines, describe the transformation method, the number of independent lines analyzed and the generation upon which experiments were performed. For gene-edited lines, describe the editor used, the endogenous sequence targeted for editing, the targeting guide RNA sequence (if applicable) and how the editor was applied.*
Authentication	*Describe any authentication procedures for each seed stock used or novel genotype generated. Describe any experiments used to assess the effect of a mutation and, where applicable, how potential secondary effects (e.g. second site T-DNA insertions, mosiacism, off-target gene editing) were examined.*

Magnetic resonance imaging

Experimental design

Design type	If not in the in-house database, a structural T1 scan was collected for neuronavigated TMS and calculation of e-field strength.
Design specifications	No functional task was run for the structural scan.
Behavioral performance measures	NA

Acquisition

Imaging type(s)	structural T1
Field strength	3 Tesla
Sequence & imaging parameters	A T1-weighted volume was acquired using an MPRAGE sequence (176 slices, whole-brain coverage, TR: 2300 ms, TE: 2.98 ms, voxel size: 1 × 1 × 1 mm, matrix size: 256 × 240 mm, flip angle: 9◦).
Area of acquisition	A whole brain scan was used.

Diffusion MRI ☐ Used ☒ Not used

Preprocessing

Preprocessing software	The structural scan was preprocessed using SPM12.

Normalization	NA
Normalization template	NA
Noise and artifact removal	NA
Volume censoring	NA

Statistical modeling & inference

Model type and settings	NA
Effect(s) tested	NA

Specify type of analysis: ☐ Whole brain ☐ ROI-based ☐ Both

Statistic type for inference<br>(See Eklund et al. 2016)	NA
Correction	NA

Models & analysis

n/a | Involved in the study
- ☒ | ☐ Functional and/or effective connectivity
- ☒ | ☐ Graph analysis
- ☒ | ☐ Multivariate modeling or predictive analysis
